# Biological Risks to Public Health: Lessons from an International Conference to Inform the Development of National Risk Communication Strategies: Report of an international conference on risk communication strategies before, during, and after public health emergencies, Rabat, Morocco, October 22-23, 2015

**DOI:** 10.1089/hs.2016.0050

**Published:** 2016-12-01

**Authors:** Petra Dickmann, Aphaluck Bhatiasevi, Fadela Chaib, Ombretta Baggio, Christina Banluta, Lilian Hollenweger, Abderrahmane Maaroufi

**Keywords:** Risk communication, Governance, Public health preparedness, Biosafety/biosecurity, International Health Regulations (2005)

## Abstract

Biological risk management in public health focuses on the impact of outbreaks on health, the economy, and other systems and on ensuring biosafety and biosecurity. To address this broad range of risks, the International Health Regulations (IHR, 2005) request that all member states build defined core capacities, risk communication being one of them. While there is existing guidance on the communication process and on what health authorities need to consider to design risk communication strategies that meet the requirements on a governance level, little has been done on implementation because of a number of factors, including lack of resources (human, financial, and others) and systems to support effective and consistent capacity for risk communication. The international conference on “Risk communication strategies before, during and after public health emergencies” provided a platform to present current strategies, facilitate learning from recent outbreaks of infectious diseases, and discuss recommendations to inform risk communication strategy development. The discussion concluded with 4 key areas for improvement in risk communication: consider communication as a multidimensional process in risk communication, broaden the biomedical paradigm by integrating social science intelligence into epidemiologic risk assessments, strengthen multisectoral collaboration including with local organizations, and spearhead changes in organizations for better risk communication governance. National strategies should design risk communication to be proactive, participatory, and multisectoral, facilitating the connection between sectors and strengthening collaboration.

Biological risks pose challenges to public health. These risks can be naturally occurring disease outbreaks at national and international levels, accidental exposure to pathogens in the context of biomedical diagnostics and research, or intentional use of pathogens for harmful purposes. Biological risk management focuses on these areas:

 • Preparing for the impact of naturally occurring disease outbreaks, on a national or international scale, on individuals and public health, national and international economies, and social and other systems;
 • *Biosafety* as understood by the UN: “principles, technologies, practices and measures implemented to prevent the accidental release of, or unintentional exposure to pathogenic agents.”^1^
 • *Biosecurity,* which refers to the “protection, control and accountability measures implemented to prevent the loss, theft, misuse, diversion or intentional release of pathogenic agents and related resources as well as unauthorized access to, retention or transfer of such material.”^1^

The World Health Organization recognized the importance of biological risks to public health and updated its International Health Regulations (IHR) in 2005 to ensure that all member states build their capacities to prevent, detect, respond to, and recover from biological and other defined risks to public health and ensure that their impact on trade and travel are minimized.^2^

Public health authorities have worked on technical areas to mitigate biological risks, such as improving disease surveillance systems and laboratory capacities.^3^ The IHR also stress the importance of risk governance in the management of public health events and have broadened the understanding of risk communication as a core capacity under the IHR (2005). While in the conventional understanding, risk communication was seen as a technical process to inform the public what to do in times of a health crisis, the current understanding of risk communication is defined by the WHO as “a multi-level and multi-faceted process, which aims to help stakeholders define risks, identify hazards, assess vulnerabilities and promote community resilience, thereby promoting the capacity to cope with an unfolding public health emergency.”^4^ This broader understanding of risk communication moves beyond a common understanding that limits risk communication to a timely conveyance of information about health risks to a public. It considers risk communication not as a technical expertise in communication but rather as a strategic activity supporting the management of public health risks by bringing in social science expertise. This transformation process thus requires national public health agencies to re-think their current risk communication strategies and plans.

In addressing this new challenge, national public health agencies have little guidance in developing their national risk communication strategies. There is some information on how to improve the communication process in a crisis^5-7^ or for particular situations, such as public health emergencies,^8^ in high security laboratories,^9^ during an influenza pandemic,^10^ or, more generally, to evaluate biosafety and biosecurity from a risk communication perspective.^11^ There is also a growing body of literature that elicits the information needs of the general public—for example, after biosecurity events^12,13^ or infectious disease outbreaks^14^—or particular at-risk groups. While these are very helpful in meeting specific communication requirements, they mostly focus on the information needs of the public and thus remain in the domain of conventional understanding of risk communication as the timely conveyance of information from experts to a lay population.

But what are the institutional and strategic steps public health authorities need to take to ensure the multilevel, multifaceted process of risk communication at an organizational level? What do public health authorities need to do and consider when developing risk communication strategies?

To tackle these questions, an international high-level conference on risk communication strategies before, during, and after public health emergencies was held in Rabat, Morocco, on October 22-23, 2015; it was organized within the framework of the German Partnership Program for Excellence in Biological and Health Security financed by the German Federal Foreign Office. The conference provided a platform to present current risk communication strategies; to learn from recent outbreaks of infectious diseases, particularly the Ebola virus disease outbreak; and to discuss recommendations with an international audience. This article summarizes the presentations and discussions from the conference and concludes with recommendations that inform the development of national risk communication strategies in Morocco and Tunisia.

## The Conference

The high-level conference was organized by the Directorate for Epidemiology (DELM) in the Moroccan Ministry of Health, together with the Deutsche Gesellschaft für Internationale Zusammenarbeit (GIZ) GmbH; it aimed to inform a project that supports the Moroccan and Tunisian ministries of health in the development of national risk communication strategies. The conference was attended by key stakeholders from Morocco, Tunisia, and Sudan and featured contributions from international experts and partners including WHO HQ, EMRO, and country office, International Federation of the Red Cross and Red Crescent Societies (IFRC), GIZ, Robert Koch Institute (RKI), and international airports.

The conference followed the logical sequence of introducing the international legislative framework (IHR and public health emergencies of international concern, or PHEIC); eliciting lessons from a recent PHEIC (the Ebola outbreak in West Africa), including the anthropological perspective from West Africa; learning from a national outbreak (Shiga toxin–producing *E. coli* in Germany); and international airports (Casablanca and Frankfurt) as points of entry, in order to stimulate thinking and discussion on risk communication strategies. A concluding panel discussed and summarized key recommendations.

## International Legislative Framework

The IHR provide the legislative framework for member states to build and strengthen the 8 core capacities, of which risk communication is one. Member states assess their capacities annually using an IHR self-assessment monitoring framework and report their assessment to WHO. More recently, the Global Health Security Agenda (GHSA), together with international partners such as WHO, have developed a joint external evaluation tool (JEE) to map and assess the strengths and weaknesses in countries’ abilities to prevent, detect, and respond to infectious disease outbreaks, with a collaborative approach using countries’ self-assessment and an assessment by external experts that results in a joint evaluation of IHR capacities.^15^

Public health events that can pose a threat to international public health can be declared under the IHR as public health emergencies of international concern. The WHO director general can declare a public health emergency of international concern following the convening of an emergency committee of experts (IHR emergency committee). The declaration of a PHEIC facilitates international coordination and collaboration in responding to and mitigating the impact of the public health emergency. The Ebola outbreak in West Africa provided some opportunities to improve risk communication, and WHO shared their reflections to inform the development of risk communication strategies.

The East Mediterranean Regional Office (EMRO) of WHO reported on a comprehensive assessment requested by the World Health Assembly in 2015 to evaluate the capacities and capabilities of the EMRO region to prevent and respond to a potential importation of Ebola virus disease into the region. This assessment identified a number of critical gaps: limited capacity for prevention, detection, and response in the areas of leadership and coordination, surveillance, infection prevention and control, risk communication, points of entry, and laboratory diagnosis. The survey revealed that risk communication was not considered essential, few had communication plans, and countries even voiced their reluctance to communicate as they felt it would create panic among the population.

These results underlined the need for raising awareness and for training to build a common understanding among health professionals and to support the development of communication strategies and plans. This assessment also revealed a discrepancy between this Ebola assessment and the IHR assessment outcome that countries report to WHO, and it reinforced the findings of the IHR monitoring review to strengthen the IHR assessment tools from self-assessment to peer review and the joint external evaluation tool.^15,16^

WHO headquarters reflected on the communication and coordination during the Ebola outbreak. The key lesson, as noted during the meeting, was that “dissemination of information alone is useless and sometimes dangerous. We must listen and constantly adjust our strategies and approaches on the basis of people's concerns.” The starting point for the risk communication process is the perception of risks in the communities, which is often different from the scientific assessment of risks. In order to gather and understand communities’ perception, interactions and engagement are necessary. This relationship-building with communities is one of the key risk communication activities. Providing technical information is only one of the building blocks, along with values, credibility, expression of caring, and, most important, trust. Trust is a key enabling factor for building relationships and engaging with communities. The IHR as a legal framework to build and strengthen the capacity in risk communication provides the context and justification to start building relationships with communities before, during, and after outbreaks.

At the height of the Ebola outbreak, many responders and stakeholders were already on the ground, and coordination proved challenging. The collaboration with international partners and, in particular, the communication with local communities provided opportunities for key insights: The dissemination of information alone (scientific facts) is not sufficient to build the relationship with local communities. In fact, a relationship and a solid degree of trust are imperative to collaborate on response and mitigation strategies and important parts of risk communication strategies that need to be integrated into the risk management process.

The example of key messages on prevention and response revealed, once again, the gap between scientific risk assessments and the public perception of risks. The scientific advice to wash hands regularly or to not touch a sick person conflicted with social realities and cultural practices in affected communities. This scientific advice was simply not applicable and integrate-able into local realities and mind sets and even offended people. To not touch a sick person was considered in communities as an unacceptable practice. This advice, though, resulted in disbelief in communities, growing distrust, and even aggression toward international and national health professionals.

Messages have to be reframed to make sense to the public. This is a process that should be informed by the scientific assessment as one source among others, such as social anthropology, psychology, and the like. The community resistance to official health advice and acceptance of rumors are, in fact, other important sources of information where risk communication has gone wrong. WHO headquarters gave an example of the relabeling of an Ebola Isolation Centre into an Ebola Treatment Centre, which helped the community to gain trust in the health facility. Rumors are particularly indicative of the strengths and weaknesses of the current risk communication process: Rumors need to be captured and deconstructed in order to improve the relationship between health workers and communities. For example, the rumor that body parts and blood were being traded was understood as a lack of trust in the health system. In a collaborative analysis of this rumor, WHO, partnering with health workers and community influencers, have reconceptualized this lack of trust. Together they reframed the approach and stressed the importance of body integrity and working with health workers and community influencers as part of the communities.

The biggest learning point for WHO was, however, the extent of community engagement and understanding communities in order to establish relevant risk communication activities. Being close, listening, and responding to the community's concern is an approach now adopted more prominently in international organizations.

### WHO Communications

Communications is an integral part of public health response and serves multiple purposes; people have the legal right to be informed about risks and how to protect themselves. The aim of communications is to enable the public to make decisions on behaviors to reduce risk. It is therefore essential to work with the media at all levels—local, national, and international—as they are quick, have a broad geographic coverage, are influential, and are often cost-effective. The WHO approach to communications during outbreak situations is characterized by 5 principles: trust, early announcement, transparency, listening (surveillance), and planning. “Showing the work, shaping the narrative” and the use of new information technology is the paradigm that WHO aims at rendering communications more proactive and transparent. This requires coordination among technical areas, understandable messages, and community engagement embedded in a listening approach.

In examples from Liberia in 2014, WHO demonstrated 3 phases of their communication approach that illustrate the organizational learning: In the initial phase, they applied a crisis communication strategy, building on the rationale of past experiences of Ebola in outbreaks in remote settings with a 90% death rate. The key messages at this early stage were that Ebola kills, Ebola has no cure, and bush meat consumption spreads the disease. These key messages were distributed via mass media, posters, and radio and resulted in denial: The perception was that Ebola spread in remote areas, and when people don't eat bush meat they won't contract the disease. It also led to sick people staying (and dying) at home as the message was that there is no cure and no treatment for Ebola.

In a second phase, the risk communication strategy moved to raising awareness that Ebola spread in major cities but with increasing survival rates. The key messages in this second phase were that Ebola is real, and there are signs and symptoms to watch that can be treated and a hotline to call for transport to treatment centers. The interventions broadened from mass media to include campaign mode using town criers and loudspeakers on trucks and motorbikes. The outcome of this awareness-raising strategy was that the demands from the public quickly exceeded response capacities and led to a lack of confidence and trust in existing structures.

In a third phase, the risk communication strategy applied community engagement with the rationale that communities are part of the solution. The key messages were to avoid unprotected contact with dead bodies, that early treatment increases survival rates, and to de-stigmatize survivors. The communication interventions were more interpersonal, with community meetings and training sessions. The outcomes of this approach were that community influencers were part of the response and that there was a need for more decentralized, localized communication.

The 3 stages of risk communication approaches during the response to Ebola were incorporated in a lessons learned process in WHO that resulted in giving more importance to community engagement and listening approaches in risk communication.

### International Federation of Red Cross and Red Crescent Societies

The International Federation of Red Cross and Red Crescent Societies (IFRC) presented a steep learning curve during its response to the outbreak. IFRC interventions in the affected region had a focus on community communication and engagement, as the people on the ground are the drivers of the disease: They can spread or stop the outbreak. Respecting local cultures, understanding their information needs, building trust, and taking time to build relationships by introducing its staff as reliable partners in a joint process of change was at the core of the IFRC approach. In addition, using new technology to improve the 2-way communication between authorities and communities and generating data on perceptions and beliefs as well as rumors were key activities to inform and shape the community engagement strategy.

There was a shift in the organization in regards to the perception of rumors: Rather than fighting misinformation, rumors were seen as important sources where more collaboration was needed. Rumors about organ trading, cannibalism, and mutilation of dead bodies in Ebola centers were circulating in the communities; Ebola was understood as a death sentence, which led to avoidance of the health centers, hiding the sick, and burying dead relatives in traditional burial rituals—which triggered more infections. Understanding rumors required a system for gathering rumors. The scarcity of information in the organization of what communities fear, know, and wish to know was a major challenge; thus, IFRC initiated a real-time emergency knowledge and information management system of socio-anthropological data and rumor management through rapid phone-based surveys building on the network of volunteers. From a governance perspective, IFRC changed their communication approach to be more inclusive and gathered regular feedback from the people and communities they worked with; this led to better relationships and, as a result, improved access to and a better understanding of the communities.

### Social Science Perspective

The social science perspective reflected the common concepts of community engagement, participative communication, and the biomedical narrative in outbreak response and advocated for a paradigm shift. Communities are smart, as their behavioral patterns and norms are survival mechanisms that make sense in their contexts. Discrediting behavior as irrational does not value the practical intelligence communities have. Rumors, for example, should not merely be understood as signs of misconception and misunderstanding but considered as important indicators that could guide outbreak detection. Fighting and “confronting” rumors with the “truth” does not work, because representatives of an official biomedical rationale and communities have different concepts of risks. While international health workers stressed, for example, the importance of hygiene and recommended regular hand washing and avoiding contact with sick or dead people, these hygiene risks were not what the communities perceived as risks. From their perspective the major risk was to be buried in a nontraditional way. In order to mitigate the risk of a nontraditional burial, communities resisted the advice and even reacted at times with violence as their worries and information demands were not met. For this reason, dialogues with communities can be unsuccessful as international health professionals represent a biomedical narrative and consider Ebola as an epidemiologic problem, whereas communities see Ebola as a social problem and may have different priorities.

The community engagement approach of international stakeholders was also critically reflected. Engaging communities is often practiced by working with key community people. However, this engagement approach is prone to replicating the official local distribution of power and authority (eg, community chiefs) and neglecting those community members who can play relevant roles but are less established in the international community engagement approach, such as faith healers, midwives, and “queen mums.” To this end, risk communication should include broader leadership roles of communities and be empowering rather than engaging.

### Institutional Reflections on Outbreak Management

The German Robert Koch Institute (RKI) continued the critical reflection on the 2 aspects of the Ebola outbreaks: Ebola as an epidemiologic event and as a social event. From an epidemiologic point of view, the RKI was prepared for an outbreak response and assessed the risk of Ebola in Germany as low. However, the public and media interest showed a dynamic that was not in relation to the epidemiologic public health risk. While the RKI was well prepared for Ebola in terms of an infectious disease, the dynamic of public awareness was somewhat surprising. Having 2 outbreak dynamics—the epidemiologic and the social dynamic—is not unique to Ebola; it is a common phenomenon.

The outbreak of Shiga toxin–producing *E. coli* (STEC), which can cause hemolytic uremic syndrome (HUS), in Germany in 2011 provided key insights into national risk and outbreak communications. This large outbreak showed a different clinical pattern and caused 54 deaths and almost 845 cases of HUS.^17^ A key challenge was to determine the source of the food contamination, and communication of this uncertainty was particularly difficult and of high impact. The public health authority found itself managing 2 challenges: the epidemiologic outbreak and the social dynamic of a strong public interest. The public health interest and media attention seemed to have followed different, nonmedical triggers, and it was thus difficult for scientists in charge of managing the outbreak investigations to integrate this dynamic into an overall management. Furthermore, certain actors from the food industry suffered economic repercussions after the announcement of false information that a vegetable from a specific country was the source of this contamination. The STEC experience showed that a better understanding of the dynamic of public interest would improve the epidemiologic preparedness planning. Risk communication strategies should therefore include the social perspective in their assessments and offer a more holistic view of the outbreak dynamics. These broader assessments require collaboration among different disciplines and technical areas and a governance approach that reinforces and encourages multidisciplinary and multisectoral collaboration in regards to more comprehensive information gathering, assessing, and sharing.

### Point of Entry: International Airports

International airports are interfaces linking the private sector of the nationally and internationally regulated aviation industry to public health, which is also regulated both at the national and international levels. To this end, airports epitomize multisectoral collaboration, with the operational rationale of minimizing negative impacts both on economies and health while maintaining business continuity. Information management and communication play key roles and are organized in standard operating procedures (SOPs) in compliance with national and international regulations. As a private sector entity, the airport is well aware of the influence of the media and aims to maintain a good relationship with journalists and other public and political groups. The key factor to success is to maintain relationships that are, at a technical level, formalized in SOPs and, at the governance level, in personal connections.

Recommendations for the development of risk communication strategies include starting risk communication early, working with multiple stakeholders, and initiating and fostering collaboration through routine information sharing and communication. Creating the governance that supports this approach is a core management task in teams and organizations dealing with risk communication.

## Discussion

The discussions at the conference revolved around 4 key areas of improvement for risk communication:

 • *Understanding communication as a multidimensional process in risk communication and enhancing listening approaches and regular feedback mechanisms to allow communities to guide and inform timely changes in risk communication strategies*

While this understanding of communication is quite well established in theory, practice and, more important, organizational governance approaches were slow to adopt this broader, interactive approach to an extent that was more than lip service. The Ebola outbreak provided a steep learning curve for organizations, with the outcome that risk communication is now incorporating community engagement and active listening in their organizational strategies at WHO, IFRC, UNICEF, and others.

 • *Broadening the biomedical paradigm and integrating social science intelligence into epidemiologic risk assessments*

Different thought cultures, scientific methods, and departments in organizations require a strong, respectful, and unifying governance approach to enable this collaboration. More important, community or lay risk assessment can challenge biomedical orthodoxies; this requires a new role for risk communication as a mediating position, offering explanations to all parties (scientists, lay people, etc) of the different concepts of risks and moderating and promoting an approach that acknowledges the differences.

 • *Strengthening multisectoral collaboration and working with local organizations*

The collaboration among professionals from different sectors and with local organizations is key to preventing and preparing for and detecting early and responding swiftly to public health events, but the collaboration can be challenging. Building this relationship requires time, patience, competence, confidence, and trust. All attributes cannot be created urgently during times of emergencies, but need to be planned for in advance and require a risk communication governance approach that promotes and reinforces this relationship building as a routine practice.

 • *Spearheading changes in one's own organization for better risk communication governance and building capacities and behavior change in one's own staff and health professionals*

Risk communication is not merely a technical capacity but a governance approach that enables broader practice and improvements of technical areas. In order to accommodate this new paradigm of risk communication, organizations need to identify and promote changes to enable this risk governance approach. Risk communication is primarily about changing one's own organizational behavior. These changes will then affect the behavior of others. To integrate this risk communication approach into the governance of organizations and national strategies is the challenge that countries and organizations will have to undertake.

## Recommendations

While these improvement areas provide important information, the key element in the discussion was how these improvements can be reflected in risk communication strategies at national levels: What do health authorities need to consider in the development of risk communication strategies? Key recommendations for the development of risk communication strategies were given in 4 areas: governance, and the 3 strategic axes of information, communication, and coordination.

***Governance and Organization***

 1. Build networks with stakeholders in the health sector and from other sectors, such as media and civil society, and start sharing information and communicating regularly.
 2. Aim for a shared community engagement approach that can be activated in case of outbreaks.
 3. Start installing a cultural change in the organization by building the competence, capacity, and skills in health authorities and professionals to understand and practice risk communication.

***Information***

 4. Integrate social science (sociology, anthropology, psychology, etc) and other aspects into risk assessment to broaden the biomedical narrative.
 5. Listen to communities to gather intelligence and regular feedback on risk communication approaches and biomedical services.

***Communication***

 6. Communication is not just the conveyance of information. Build relationships and engage with communities and media.
 7. Media should be seen as a partner in supporting risk communication to bridge the gap between the perception of the public and the scientific assessment of risks.
 8. Have a proactive, transparent, and participatory communication approach.

***Coordination***

 9. Have clear and transparent coordination and collaboration mechanisms that enable learning.
 10. Integrate risk communication into public health disciplines to improve risk assessment, planning, and preparedness for public health risks.
 11. Have a national risk communication strategy and operational plan including all stakeholders—for instance, local organizations working at the community level; share nationally and internationally and exercise this approach.

## Conclusion

The conference provided a platform for exchanging experiences and expertise, and the discussion highlighted the major themes of risk communication: Risk communication should be proactive, participatory, and multisectoral, facilitating the connection between sectors and strengthening collaboration. National strategies on risk communication should conceptualize risk communication on governance and organizational levels and along its 3 strategic axes of information, communication, and coordination. Relevant areas for improvement were identified, including understanding communication as a multidimensional process in risk communication, broadening the biomedical paradigm and integrating social science intelligence into epidemiologic risk assessments, strengthening multisectoral collaboration to ensure relevant advice, and spearheading changes in one's own organizations to improve risk communication governance and develop capacities and behavioral change in one's own staff and health professionals. The results of this conference inform the development of national risk communication strategies in Morocco and Tunisia.
